# The Perceived Impact of the New Medicare Rules for Split/Shared Visits: A Survey of Advanced Practice Administrators

**DOI:** 10.7759/cureus.40815

**Published:** 2023-06-22

**Authors:** Vasco Deon Kidd, Jennifer Hammonds

**Affiliations:** 1 Department of Orthopedic Surgery, University of California, Irvine, School of Medicine, Orange, USA; 2 Office of Compliance and Privacy, University of California, Irvine, Orange, USA

**Keywords:** time-based billing, advanced practice providers, medical compliance, cms guidelines, physician assistant, physician associate, advance practitioner nurse, split/shared, medical billing, medicare

## Abstract

Background

The Centers for Medicare and Medicaid Services (CMS) recently updated its split/shared policy but delayed enforcing sole time-based billing methodology in 2023 after industry pushback. However, the new time-based requirement is set for 2024. Yet, there is no published literature addressing perceived organizational impacts associated with new split/shared rules.

Methods

A cross-sectional survey study was administered via electronic email Listserv (n = 108) over a two-and-a-half-week period in 2023. The survey was conducted to examine the potential organizational impact of complying with the new Medicare split/shared visit policy. The collected survey data were analyzed using descriptive statistics.

Results

Despite the small sample size, this novel research study seems to suggest that there is a range of perceived issues associated with the new split/shared rules, including a perceived decrease in physician compensation due to changes in work relative value unit (wRVU) attribution and potential conflict between physicians and advanced practice providers (APPs) as they compete for wRVU credit for inpatient services. Additionally, respondents felt that the new regulatory change would lead to layoffs and/or hiring freezes of inpatient APP positions, thereby impacting team-based care dynamics. Further work is needed to better understand the impact of the new split/shared rules on coding practices, revenue, efficiency, provider, and patient satisfaction.

Conclusion

The findings reported in this study shed new light on the perceived impacts of the new split/shared visit rules on healthcare institutions. Although the updated rules are designed to provide greater transparency and better align reimbursement with services performed, concerns persist around the potential impact on day-to-day workflows, physician compensation, net revenue, and potential economic impact on the traditional team-based care model.

## Introduction

Physician assistants/associates (PAs) and nurse practitioners (NPs) collectively known as advanced practice providers (APPs) practice in all 50 states and in a variety of different specialties within the United States and abroad. Although there are differences in certification, educational philosophy, and licensure, both NPs and PAs perform overlapping responsibilities in the clinical setting, including billing for both clinical and procedural services [1-5]. With regard to hospital inpatient billing, APPs and physicians treating patients jointly have been subject to split/shared billing rules since October 2002; however, the Centers for Medicare and Medicaid Services (CMS) recently made substantive modifications to their split/shared evaluation and management (E/M) visit policy. Before discussing the policy change, it is important to provide a historical timeline chronicling events that contributed to the evolution of split/shared billing services in the United States. Prior to 1997, in the facility setting (hospital inpatient, observation care, emergency department, outpatient hospital, etc.), CMS recognized nurse practitioners (NPs) and physician assistants (PAs) as facility support staff. APP services were reimbursed through the hospital’s annual cost report, and no Part B billing was allowed. However, the Balanced Budget Act of 1997 amended Section 1861(b)(4) of the Social Security Act, allowing APPs to be recognized as Part B providers, who would be required to obtain a national provider identifier (NPI) and enroll in Medicare. At that time, it was determined that APP services would be reimbursed at 85% of the Medicare Physician Fee Schedule (MPFS).

Although recognizing APPs as Part B providers in the facility setting was a significant advancement in APP clinical practice, the financial implications were substantial as APP salaries could no longer be included in the hospital’s cost report. To help offset the cost, CMS developed the long-standing practice of split/shared billing, which many commercial payers adopted.

The split/shared practice allows for E/M services, jointly performed by a physician and APP, to be billed at 100% of the MFPS, under the physician’s name and NPI number, when all billing requirements are met. Historically, most split/shared services were billed under the physician’s name with few requirements regarding the amount of physician participation or the level of documentation needed. As part of CMS’s annual rulemaking process, the split/shared guidelines were updated in 2022.

The new rule states that billing should be attributed to the provider (physician or APP) who spent the substantive portion of time, which is defined as greater than 50%, in the care of the patient on that calendar day. The rule also allows critical care services and certain skilled nursing facility services (SNFs) to be split/shared and mandates a billing modifier “FS” to be appended to all split/shared services. The FS billing modifier will allow Medicare to be able to readily identify shared services creating additional scrutiny and targeted payer auditing.

The implementation of these changes began in 2022 and carried over into 2023, which is labeled as a transitional year. While critical care services, including split/shared critical care, are solely time-based, non-critical care services can now be attributed either to time or the performance of history, examination, or medical decision-making (MDM). Since history and examination are no longer used to level-set E/M codes, this language is a bit nebulous. Anecdotally, during this transitional year, most groups have chosen to document and bill under an MDM rubric for inpatient E/M visits.

Under the MDM rubric, non-critical care split/shared visits are generally billed by physicians and are being reimbursed at 100% of the MPFS, assuming that all billing requirements are met. However, the billing requirements are not clearly outlined and have led to confusion among healthcare institutions and others [6]. For example, to bill, there must be a face-to-face visit by either the physician or the APP; however, the current rule does not require the billing provider to be the one who performed the face-to-face portion of the visit. The combined documentation of the APP and physician may be used to support the services billed; however, the rules do not outline what level of documentation is expected by the physician or APP. Lastly, both the physician and APP must be employed by the same group.

In 2024, CMS plans to move to a solely time-based attribution model. Although the 2022 final rule included several changes that are more in line with current clinical practice and recognize APPs’ expanding roles as members of the care team, the impact of those changes on the team-based care model and revenue expectations is yet to be determined.

Research aim

Given this background and little to no published research relevant to this topic, the current study was undertaken to explore the perspectives of advanced practice provider administrators/leaders on the perceived organizational impact of the new split/shared policy including the possibility of transitioning to a solely time-based billing methodology in 2024.

## Materials and methods

University of California, Irvine, issued approval with IRB #3044 self-exempt. An 18-item cross-sectional descriptive anonymous survey was developed by the study investigators. The survey included primarily multiple-choice and some multi-select questions. The survey was built using the Qualtrics survey platform (Qualtrics Inc., Provo, UT, USA). The survey questions were piloted with three content experts from different institutions with expertise in inpatient and outpatient coding and billing compliance. Expert opinion resulted in minor changes to some of the questions, which improved the overall quality of the survey. The survey was finalized and disseminated online through an established healthcare administrative Listserv of advanced practice administrators. Advanced practice administrators were surveyed due to their expertise in APP roles and scope of practice considerations, credentialing and privileging, business case development, recruitment and retention efforts, and responsibility for revenue cycle functions of APP split/shared billing. Three emails bounced back or failed to deliver, resulting in a total of 105 valid emails. The email introduction contained the purpose of the study including all the necessary elements of written consent, and submission of the survey indicated the respondents’ consent to participate. Confidentiality was maintained throughout the study. The study period was from April 2023 to May 2023. A total of nine email reminders were sent to study participants to encourage survey participation. Prepaid incentives were not used to improve response rates. The average length of time to complete the survey was less than five minutes. The survey used a skip logic methodology, allowing participants to skip certain questions based on responses to preceding survey items. Descriptive statistics were used to summarize the data. When calculating sample size, it is estimated that 83 (79%) or more survey responses are needed to have a confidence level of 95% within a 5% margin of error.

## Results

Twenty-eight participants responded to the survey. Three participants submitted incomplete surveys where less than 50% of the total items were completed. The partially completed surveys were not included in the data analysis. The overall response rate is 23.8% (25/105). Of those that completed the survey, 56% (14/25) were nurse practitioners (NPs), 40% (10/25) were physician assistants (PAs), and 4% (1/25) were compliance officers. The distribution of respondents with respect to job title included director of advanced practice providers (52%, 13/25), chief of advanced practice (12%, 3/25), vice president of advanced practice (8%, 2/25), senior director of advanced practice providers (4%, 1/25), interim director of advanced practice providers (4%, 1/25), executive director of advanced practice providers (4%, 1/25), manager of advanced practice providers (4%, 1/25), senior vice president of advanced practice (4%, 1/25), chief compliance officer (4%, 1/25), and advanced practice provider administrator (4%, 1/25). The majority of the respondents (64%, 16/25) are located in an academic medical center (AMC), while 36% (9/25) are located in a multiple hospital system.

Role of advanced practice providers

**Figure 1:**
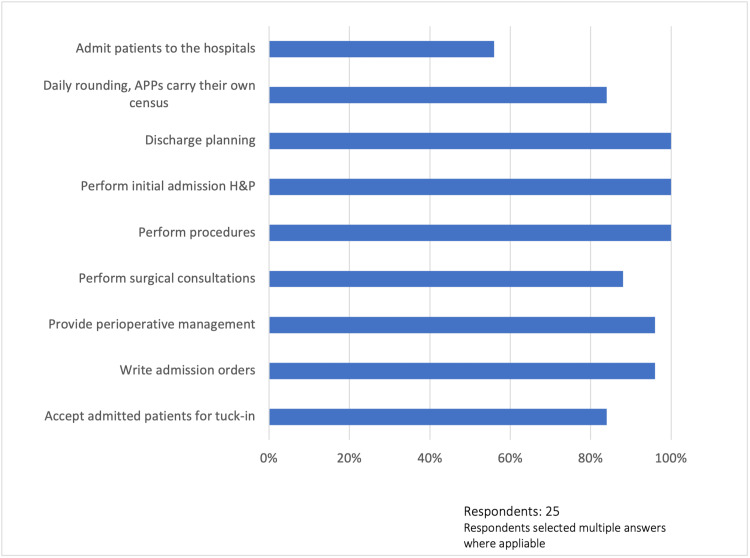
Role of advanced practice providers APPs: advanced practice providers, H&P: history and physical examination

The respondents indicated that the role functions of advanced practice providers included initial admission history and physical examination (100%, 25/25), discharge planning (100%, 25/25), procedures (100%, 25/25), writing admission orders (96%, 24/25), providing perioperative management (96%, 24/25), surgical consultations (88%, 22/25), accepting admitted patients for tuck-in (84%, 21/25), daily rounding with APPs carrying their own census (84%, 21/25), and admitting patients to the hospital (56%, 14/25). These clinical activities are summarized in Figure 1.

Work relative value unit (wRVU) expectations

**Figure 2:**
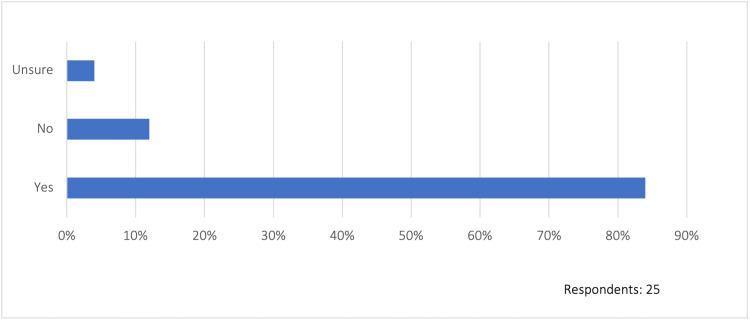
Do physicians receive wRVU credit from APP-generated work? wRVU: work relative value unit, APP: advanced practice provider

Of the respondents, 48% (12/25) indicated that their organization has established set wRVU targets for independent APP visits (where no physician participation is required), while 48% (12/25) do not. Additionally, 88% (22/25) of the respondents indicate that there is a productivity wRVU-based component to physician compensation at their organization. In fact, 84% (21/25) of the respondents indicate that their physicians employed on a productivity-based compensation model receive credit for APP-generated wRVUs (Figure 2). However, it is unclear from the data whether the wRVU credit received by physicians is tied to how it was billed either in the APP’s name or split/shared.

Inpatient billing practices

**Figure 3:**
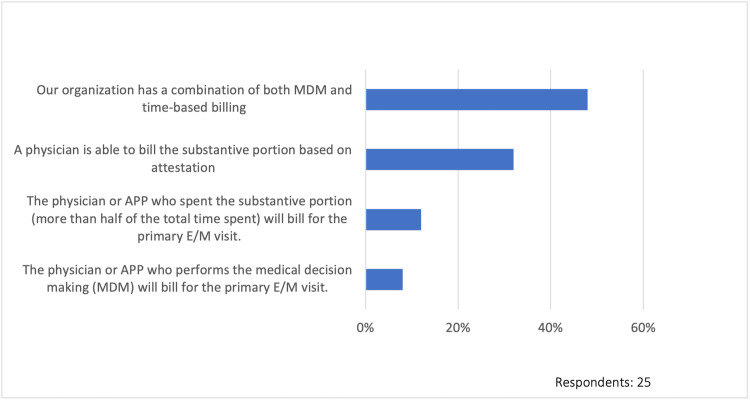
Current inpatient split/shared billing practice MDM: medical decision-making, E/M: evaluation and management services

To understand the billing practices among healthcare organizations, we asked a series of questions. Nearly half of the respondents (48%, 12/25) indicate that their organization uses a combination of both medical decision-making (MDM) and time-based billing practices. Although 32% (8/25) report that a physician is able to bill the substantive portion based on attestation, 12% (3/25) indicate that the physician or APP who spent the substantive portion (more than half of the total time spent) will bill for the primary evaluation and management (E/M) visit. About 8% (2/25) stipulate that the physician or APP who performs the medical decision-making (MDM) will bill for the primary E/M visit (Figure 3).

Time-based billing

**Figure 4:**
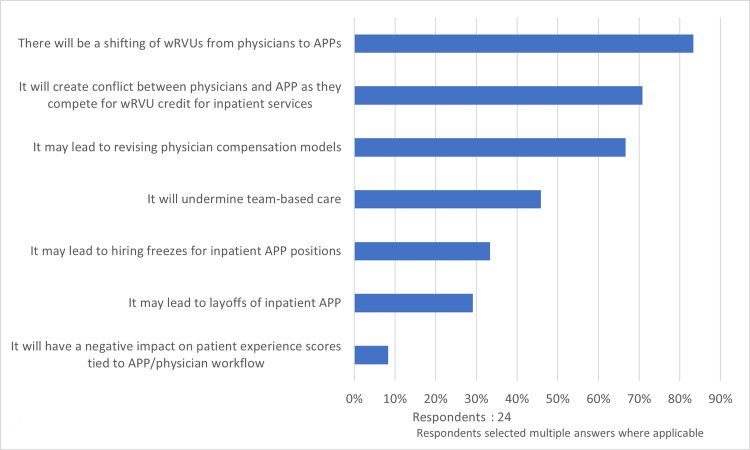
Anticipated impact of time-based billing wRVU: work relative value unit, APP: advanced practice provider

The respondents were probed using a multi-select question regarding their perceptions about the organizational impact of converting to a time-based billing-only methodology. Although most respondents surveyed have not fully converted over to time-based billing, 44% (11/25) indicate that their organization is in the process of transitioning completely over to time-based billing for all inpatient services involving split/shared encounters. The concerns expressed by the respondents regarding split/shared time-based billing included a shifting of wRVUs from physicians to APPs (83%, 20/24) and conflict between physicians and APPs as they compete for wRVU credit for inpatient services (71%, 17/24). Other potential impacts included revising of physician compensation models (67%, 16/24), undermining of team-based care (46%, 11/24), hiring freezes of inpatient APPs (33%, 8/24), layoffs of inpatient APPs (29%, 7/24), and a negative impact on patient experience scores tied to APP/physician workflows (8%, 2/24) (Figure 4). One respondent reported that it may lead to hiring freezes for inpatient physicians (4%, 1/24).

Perceived barriers in tracking APP inpatient billing activity

**Figure 5:**
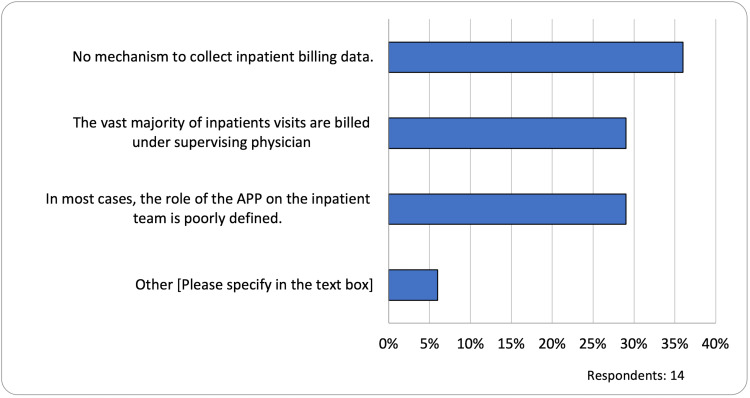
Barriers to tracking inpatient APP productivity APP: advanced practice provider

The reported barriers to tracking APP inpatient billing activity included no mechanism to collect inpatient billing (36%, 5/14), the vast majority of inpatients visits being billed under the supervising physician (29%, 4/14), and, in most cases, the role of APP on the inpatient team is poorly defined (29%, 4/14) (Figure 5).

Some selected respondent quotes regarding the new split/shared visit rules are shown in Table 1. 

**Table 1:** Respondent quotes regarding the new split/shared visit rules APP: advanced practice provider

Selected respondent quotes
“It will lead to challenging workflow implementation and the possibility of using a macro or templating documentation to meet time standards.”
“It may lead to re-allocation of APPs within APP-physician patient care teams.”
“(It) will lead to better documentation and capture of work being done and by whom.”
“This is actually a positive impact as it is allowing us to move to more APP-independent billing practices and relooking at the APP models of care we have at our organization.”
“This standard is requiring substantial resources to train, implement, and track. We continue to face pushback on implementing the standard mostly due to not wanting to change current workflow and billing practices and question why they need to on the inpatient floor, (and) this will possibly lead to workarounds to maintain the current practice.”

Outpatient facility billing practices

**Figure 6:**
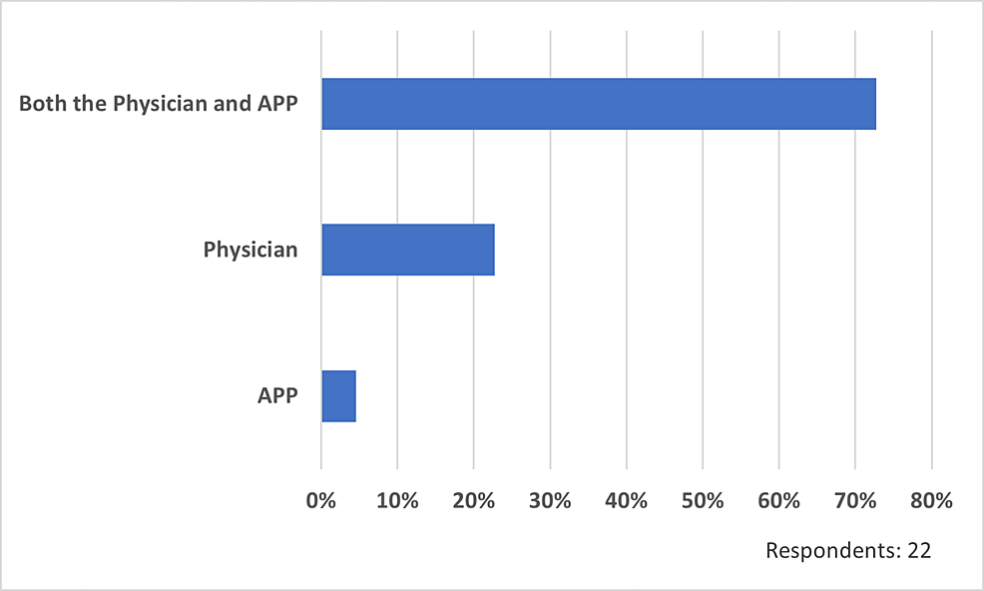
Who is responsible for medical decision-making? APP: advanced practice provider

Of the respondents, 73% (16/22) indicate that both the physician and APP are responsible for the medical decision-making (MDM) in split/shared encounters in the outpatient facility setting (excluding place of service 11) compared to physician only (23%, 5/22) or APP only (5%, 1/22) (Figure 6). Additionally, the respondents report that their hospital routinely tracks split/shared visits in the outpatient facility and inpatient setting (73%, 16/22), while 27% (6/22) track only inpatient split/shared visits.

Electronic medical record capture

**Figure 7:**
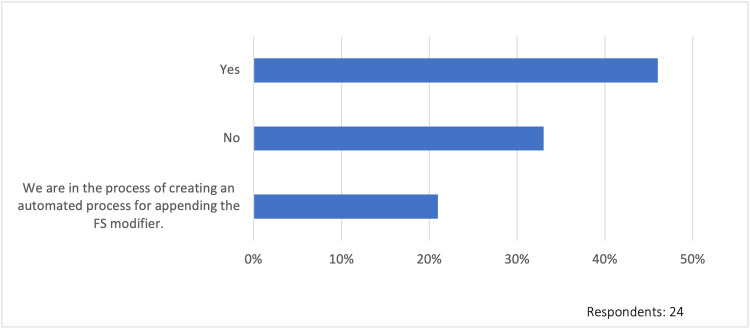
Automated process in the electronic medical record to append FS modifier

Only 42% (10/24) of the respondents indicated that their electronic medical record (EMR) is set up to capture the amount of time spent by physicians and APPs, while 12% (3/24) were unsure if their EMR had this capability. Moreover, 46% (11/24) indicated that they have an automated process in their electronic health record that appends the modifier FS to facility claims for split/shared visits, whether the physician or APP bills for the visit. Furthermore, 21% (5/24) indicated that they are in the process of creating an automated process for appending the “FS” modifier. The findings related to EMR capture are summarized in Figure 7.

## Discussion

To our knowledge, this is the first study to date to investigate the perceived organizational impact of the new split/shared visits rules through the lens of APP administrators and leaders within the United States. Unsurprisingly, this study’s findings indicate that APPs perform a myriad of clinical activities in the hospital setting, such as completing the initial admission history and physical examination, writing admission orders and admitting patients to the hospital, and accepting admitted patients for tuck-in. The respondents further reported that APPs provide perioperative management, conduct surgical consultations, perform procedures, perform daily rounding while carrying their own complement of patients, and are involved in discharge planning, which is consistent with previous research [7-9]. It is unknown whether APPs were performing some of these clinical tasks independent of physician supervision. Nevertheless, the findings demonstrate the versatility inherent in the APP role as these providers are uniquely qualified to address the specific needs of patients. In fact, emerging data demonstrate that utilizing APPs to top-of license improves bottom-line expectations, patient access, patient satisfaction, and provider engagement without jeopardizing physician productivity [2,3].

Additionally, our study demonstrates that the respondents were concerned about the effects of the new split/shared rules on their healthcare organizations. Perceived financial impacts included shifting of wRVUs from physicians to APPs and competition for wRVUs between physicians and APPs leading to potential downstream effects on physician compensation and incentive models. This may have unintended consequences on the physician and APP team dynamic as the respondents reported that physicians employed on a productivity-based compensation model receive credit for APP-generated wRVUs. Other potential impacts reported by the respondents included layoffs and/or hiring freezes of inpatient APP positions, which may potentially impede the growth of team-based care models and may lead to a more APP-centric model (lower costs) or a physician-centric model (higher reimbursement). Despite these findings, it remains unclear how significant the effects of the new rules will be on healthcare institutions, but concerns exist, nonetheless.

While sufficient documentation is key to proper reimbursement, reduced malpractice risk, and quality clinical care, the new split/shared rules are difficult to demystify and may create unnecessary confusion and administrative burden for healthcare systems. For example, physicians are required to perform, but not explicitly document, history, examination, or MDM; however, history and examination are no longer used to level-set E/M codes. Furthermore, is there an expectation that physicians must document their work? If not, then are physicians able to demonstrate compliance by documenting a single element from history, examination, or MDM and attesting to the APP’s progress note? Although a significant number of respondents are making technological changes in their systems to accommodate the new split/shared rules, further clarification of the new rules may result in rework, additional costs, and disruption to existing technology-supported processes.

As we move into a split/shared time-based billing model, there are several compliance issues that will need to be monitored, including the over-documentation of time, leading to an increase in unbelievable days. Unbelievable days occur when a clinician bills for more hours than they could have possibly worked. And while not all providers bill based on time, each E/M code carries an associated time value that is used to identify data outliers and improper billing.

It is believed that CMS is looking to improve visibility into APP-driven clinical quality and/or improved attribution and visibility into individual productivity. This is to ensure that the pay is commensurate with the work performed, and if the APP is performing the substantive portion of the visit, then the visit should be billed at the 85% rate. In the 2022 MPFS Final Rule, Split or Shared, Defining Substantive Portion, C, CMS stated, “We did not believe it would be appropriate to consider a brief or minor interaction, with or without direct patient contact, such as where the physician merely ‘pokes their head’ into the room, to be a substantive portion of the visit” [10]. While we understand CMS’s goal with time-based billing, it remains to be seen whether the new changes will ensure payment is made at the correct reimbursement rate.

Although the new CMS split/shared policy represents a substantial sea change, there remain many unanswered questions. For example, when time-based billing becomes mandatory, will CMS clarify what the physician is required to document in order to support the time they personally spent with the patient? Will CMS expand upon the billing provider not being required to personally perform the face-to-face? Will CMS clearly define what a medically necessary split/shared service is? Without clear guidance around expectations, split/shared billing may likely see an uptick in abuse that may carry massive penalties for failing to comply. Part of the uptick in abuse may stem from a lack of clear guidance by CMS around the new rules and the fact that some physicians may rely too heavily on wRVU credit generated by the APP’s work. To avoid potential fraud risk, physicians should not rely on APP-generated wRVUs.

There is already a significant focus on both the split/shared and incident to billing practice as evidenced by the most recent False Claims Act settlement from May 9, 2023. Yale New Haven Health Services Corporation and Northeast Medical Group entered into a civil settlement agreement with the federal and state governments to pay $560,718.48 to resolve allegations that they violated the federal and state False Claims Acts by billing services under the physician’s name, when in fact the services should have been billed by advanced practice registered nurses and physician’s assistants at the lower reimbursement rate [11].

Lastly, the implementation of the new FS modifier is an indicator that CMS wants visibility into the practice of split/shared and will continue to audit healthcare organizations for compliance. Therefore, it is important for healthcare institutions to create a viable model to ensure that split/shared visits are billed according to the new guidelines without hindering team-based patient care. ​​​​

Limitations

Despite the many strengths of this rare study, there were some limitations. The main limitation of the study is a relatively low response rate despite multiple email reminders to complete the survey. Other studies using email Listserv have also reported low response rates [12,13]. The present study’s response rate limits the generalizability of our findings, and thus, selection bias may be present. Given the anonymous nature of the survey, demographic data of non-responders was not available for analysis. Additionally, this research was limited mainly to advanced practice provider administrators. Therefore, caution is needed in interpreting the results of this study.

Areas of future research

Our study results serve as a stepping-stone to future research. Additional investigation is required to explore the impact of split/shared time-based billing on the physician and APP care model along with productivity differences between inpatient physicians and APPs. Another area of research is to evaluate the impact of the split/shared criteria regarding physician compensation arrangements and financial performance incentives. Lastly, although our study did not examine the impact of these new split/shared rules on longstanding APP inpatient training fellowships/residencies, future research should be undertaken to investigate the variation in billing patterns between licensed APP postgraduate trainees and attending physicians [4,14,15].

## Conclusions

This study seems to suggest that the new split/shared billing rules will undoubtedly have long-lasting effects on healthcare organizations, including a perceived economic impact on traditional team-based care models. However, additional research with a larger sample size is needed to further examine the full impact of the new changes on healthcare institutions. As CMS looks to reduce their expenses and ensure appropriate attribution, the 15% payment difference between physicians and APPs represents low-hanging fruit. However, reduced reimbursement under the APP may lead to a degradation of revenue, which may in part be mitigated by loosening physician supervision requirements, thereby freeing up physicians to concentrate on additional revenue-generating activities. Lastly, healthcare organizations will need to develop strategies to address the potential perceived consequences such as workplace conflicts around compensation and potential disruption to wRVU credit that may arise within healthcare teams because of the new split/share rules.

## References

[1] Vasco Deon K (2020). Redeployment of orthopaedic advanced practice providers at academic medical centers during the COVID-19 pandemic. Orthop Nurs.

[2] Kidd VD, Liu JH, Reamer-Yu A, Wang JH, Deng M (2022). The development of a visual dashboard report to assess physician assistant and nurse practitioner financial and clinical productivity. BMC Health Serv Res.

[3] Kidd VD, Amin A, Bhatia N (2023). Optimal use of advanced practice providers at an academic medical center: a first-year retrospective review. Cureus.

[4] Kidd VD, Vanderlinden S, Hooker RS (2021). A national survey of postgraduate physician assistant fellowship and residency programs. BMC Med Educ.

[5] Katz J, Powers M, Amusina O (2021). A review of procedural skills performed by advanced practice providers in emergency department and critical care settings. Dis Mon.

[6] (2023). CMS urged to rescind APP split/shared policy. https://www.chestnet.org/Newsroom/CHEST-News/2022/04/CMS-urged-to-rescind-APP-split-shared-policy.

[7] Hickman D (2023). The role of NPs and PAs in hospital medicine programs. https://www.the-hospitalist.org/hospitalist/article/142565/leadership-training/role-nps-and-pas-hospital-medicine-programs/2/.

[8] Kleinpell RM, Grabenkort WR, Kapu AN, Constantine R, Sicoutris C (2019). Nurse practitioners and physician assistants in acute and critical care: a concise review of the literature and data 2008-2018. Crit Care Med.

[9] Shannon EM, Cauley M, Vitale M (2022). Patterns of utilization and evaluation of advanced practice providers on academic hospital medicine teams: a national survey. J Hosp Med.

[10] (2022). MPFS final rule, split (or shared) visits: C: Definition of substantive portion. https://www.federalregister.gov/d/2021-23972/p-1243.

[11] (2023). Attorney General Tong announces $560,718 false claims settlement with hospital owner and medical group. https://portal.ct.gov/AG/Press-Releases/2023-Press-Releases/Attorney-General-Tong-Announces-False-Claims-Settlement-with-Hospital-Owner-and-Medical-Group..

[12] Yong M, Mijovic T, Lea J (2016). Endoscopic ear surgery in Canada: a cross-sectional study. J Otolaryngol Head Neck Surg.

[13] Hoch AM, Schoenberger SF, Boyle TP, Hadland SE, Gai MJ, Bagley SM (2022). Attitudes and training related to substance use in pediatric emergency departments. Addict Sci Clin Pract.

[14] Kidd VD (2023). Exploring motivations and barriers to accreditation adoption among physician assistant and nurse practitioner emergency medicine and orthopedic surgery residency and fellowship programs. Cureus.

[15] Klimpl D, Franco T, Tackett S, Cardin TE, Wolfe B, Wright S, Kisuule F (2019). The current state of advanced practice provider fellowships in hospital medicine: a survey of program directors. J Hosp Med.

